# Concordance of sibling's recall of measures of childhood socioeconomic position

**DOI:** 10.1186/1471-2288-11-147

**Published:** 2011-11-01

**Authors:** Michael M Ward

**Affiliations:** 1 Intramural Research Program, National Institute of Arthritis and Musculoskeletal and Skin Diseases, National Institutes of Health, Bethesda, MD, USA

**Keywords:** Education, Occupational groups, Childhood socioeconomic position

## Abstract

**Background:**

Studies of socioeconomic determinants of health often rely on recalled information on childhood socioeconomic position, despite limited evidence of the validity of this information. This study examined concordance between siblings of recalled measures of childhood socioeconomic position.

**Methods:**

This cross-sectional study examined reports by 1280 adult sibling pairs in the National Survey of Midlife Development in the United States of seven measures of childhood socioeconomic position: father's occupation (in 9 categories), father having a professional occupation, father being a supervisor at work, father's education level, mother's education level, receipt of welfare payments, and subjective appraisal of being better or worse off financially than others.

**Results:**

Concordance was high for father's professional occupation (0.97; 95% confidence interval (CI) 0.96, 0.98), father's occupation in 9 categories (0.76; 95% CI 0.73, 0.80), and receipt of welfare payments (0.95; 95% CI 0.93, 0.97). Concordance was lower for father's and mother's education level, and lowest for subjective appraisal of socioeconomic position (0.60; 95% CI 0.57, 0.64). Concordance of parental education was lower for sibling pairs with high school educations or less.

**Conclusion:**

Concordance of recalled measures of childhood socioeconomic position by siblings is generally but not uniformly high.

## Background

Studies of socioeconomic determinants of health have increasingly adopted a life-course perspective, examining associations not only of current socioeconomic position but also of measures of childhood socioeconomic position [1,2]. Although some longitudinal cohort studies have collected data on childhood socioeconomic position prospectively, this approach is often not feasible in clinical and epidemiological studies. Most studies assess childhood socioeconomic position retrospectively using recall by study participants [3-5].

Few studies have assessed the validity of recall of measures of childhood socioeconomic position. In a British survey, the occupation of the subject's father was accurately recalled after 50 years by 12 of 18 subjects, but recall was assessed by a detailed interview that first established personal life histories [6]. A larger British study reported that childhood social class, based on the father's occupation, was accurately recalled by 54% of adults when compared to prospectively collected data [7]. In contrast, Krieger et al reported highly concordant responses to questions on father's educational attainment and work role by 352 adult twin pairs, indicating that these measures were accurately recalled [8]. The study included only women, and examined only two measures of childhood socioeconomic position, leaving open the question of whether recall would be similarly concordant among men and for other measures of childhood socioeconomic position. Although father's occupation and father's education level have been the measures most often used to assess childhood socioeconomic position, other measures such as mother's education level, indicators of poverty or financial insecurity, and subjective appraisal of relative wealth have been included in recent surveys [3,9,10]. The goal of this study was to assess the validity of recall by adults of several different measures of childhood socioeconomic position by comparing responses between sibling pairs in a large national sample. We also examined if concordance varied with characteristics of the respondents.

## Methods

### Source of data

Data were from the National Survey of Midlife Development in the United States (MIDUS), a survey of the health, psychological well-being, and social and economic circumstances of adults in mid-life in the United States (U.S.), conducted in 1995-1996 [11]. The survey included four samples: the main sample, siblings of participants in the main sample, a sample of twins, and dedicated samples in five selected cities. Participants in the main sample were recruited by random-digit dialing of households in the contiguous U.S. from working telephone banks to provide a representative sample of noninstitutionalized English-speaking adults. One member of each household who was age 25 to 74 was randomly selected to participate. Participants completed a telephone interview and were then asked to complete a mailed self-administered questionnaire. The response rate for telephone interview was 70% and for the questionnaire was 87%, for a final sample of 3032 participants. Siblings were recruited from a random subsample of 529 participants in the main sample who completed the interview. Only full siblings age 25 to 74 were eligible, but more than one sibling per family could be enrolled. Interviews were completed by 950 of 1480 siblings identified. Twins were referred from other national surveys that screened approximately 50,000 households for the presence of twins, of which 60% were enrolled (n = 1914). Some families had more than one twin pair enrolled, and for some pairs, both twins did not enroll. The city-specific samples were not included in this study.

From these samples, we identified 529 sibling pairs and 885 twin pairs, selecting at random one twin pair per family in cases where more than one twin pair had been enrolled. For families with more than one sibling enrolled, we selected the sibling closest in age to the participant in the main sample. One sibling pair per family was selected to avoid non-independent observations. Of these 1414 pairs, we then excluded 134 pairs for which either the identified male head of household during most of their childhood (specified as before age 17) or the identified female head of household during most of their childhood differed between members of the pair. For example, one member of the pair may have identified the biological father as the male head of household while the other member of the pair identified a step-father as the male head of household. Because information on education and occupation were specifically asked about the head of household, we required both members of each pair to reference the same individual. Eighty-six pairs were deleted because they identified different male heads of household, 32 pairs were deleted because they identified different female heads of household, and 16 pairs were deleted because both heads of household differed, resulting in 1280 pairs (476 sibling pairs and 804 twin pairs).

### Measures of childhood socioeconomic position

Questions on measures of socioeconomic position before the age of 17 were asked during the telephone interview. Participants were asked to report the main job title of the male head of household (hereafter, father), which survey investigators then classified into one of nine categories of the U.S. census occupational classification system (professional, manager, technical worker, clerical, sales, craftsman, service worker, operative/laborer, farm worker) [12]. For analysis, the father's occupation was considered both as the 9-category classification and as a dichotomous variable representing professional occupation versus other. Data were only collected for a single main job title; if respondents reported their father changed jobs, they were instructed to report the main job he had during their adolescence. Participants were also asked if their father supervised others at work. Participants were asked their father's highest level of educational attainment in 12 categories, which for analysis was collapsed into 5 categories (grade school, some high school, high school graduate or General Educational Development qualification, some college, and college graduate). Educational attainment of the female head of household (hereafter mother) was similarly classified. Participants were asked if during their childhood or adolescence their family had received welfare or Aid to Dependent Children for at least 6 months. Lastly, participants were asked if they thought that while growing up, their family was better off or worse off financially than other families at the time, on a 7-category scale ranging from "a lot better off" to "a lot worse off". For analysis, responses were collapsed into three categories (better off, the same, and worse off). Only 28.6% of participants reported that their mother worked during most or all of their childhood, so mother's occupation was not analyzed.

### Data analysis

Percent concordant responses between members of each pair were tabulated for each measure of childhood socioeconomic position, with 95% confidence intervals based on binomial proportions. Concordance measures only identical responses and does not account for chance. Agreement was therefore also estimated using weighted kappa, with exact 95% confidence intervals. Kappa provides a measure of agreement beyond that expected due to chance alone. Weighting of the kappa takes account of the degree of discrepancy between ordinal responses, with widely divergent responses discounted more than slightly divergent responses. However, kappa is sensitive to the prevalence of responses across categories [13]. Higher kappa indicates greater agreement. Data were missing for at least one member for father's occupation in 142 pairs (11.1%), for father's supervisory role at work in 126 pairs (9.8%), for father's education level in 174 pairs (13.6%), for mother's education level in 99 pairs (7.7%), for welfare during childhood in 20 pairs (1.5%), and for subjective appraisal of whether the family was better or worse off financially than others in 126 pairs (9.8%). These pairs were excluded from the corresponding concordance estimate because only non-missing responses are informative for concordance. Data were missing for both members of the pair for between 10% (welfare during childhood) and 38% (father's supervisory role at work) of pairs with missing data. No pairs had missing data on all measures.

To investigate if the degree of concordance was related to participant characteristics, we computed estimates for subgroups by age (younger or older than the group median of 46 years, and categorized based on the age of the younger member of the pair), sex, twin status, education level (less than high school, high school graduate, some college, or college graduate, based on the education level of the member of the pair with the lowest education level), and income (poor versus not poor). Pairs were classified as poor if either member reported an annual household income of less than $31,200, which was 200% of the 1996 federal poverty level for a family of four. Adjustment of income for household size was not possible because data on the number of members in the household was not available.

Analyses were performed using SAS programs (SAS Inc, Cary, NC).

## Results

The sample included 2560 participants (1280 pairs), of whom 44.6% were men and 89% were white; 36.3% had a high school education or less (Table 1). The age difference between siblings was 4 years or less in 71.4% of non-twin pairs. Brothers comprised 26.8% of pairs, sisters comprised 37.6% of pairs, and a brother and sister comprised 35.6% of pairs. Ninety-three percent of pairs reported on both of their biological parents.

**Table 1:** Characteristics of siblings in the National Survey of Midlife Development in the United States (N = 2560).

Age, years *	46.7 ± 12.5
Women, n %	1419 (55.4)
White, n %	2282 (89.1)
Black, n %	54 (2.1)
Other, n %	224 (8.8)
Education < high school graduate, n %	188 (7.3)
High school graduate, n %	743 (29.0)
Some college, n %	772 (30.2)
College graduate, n %	857 (33.5)
Household income, dollars ^†^	60,000 (33,500 - 100,500)
Twin, n %	1608 (62.8)
Reported on biological father, n %	2388 (93.3)
Reported on biological mother, n %	2514 (98.2)

* Mean ± standard deviation

^† ^Median (25^th^, 75^th ^percentile)

Concordance for father's occupation, based on the 9-category classification, was 0.76 and kappa was 0.77, indicating substantial agreement (Table 2). Concordance was higher when considering only whether the father had a professional occupation or not, ignoring discrepancies in other categories of occupation. Concordance for father's supervisory role at work, father's education level, and mother's education level was slightly lower, ranging from 0.69 to 0.77, but had substantial agreement within pairs. There was high concordance on whether the family had received welfare payments for 6 months or longer. Kappa for welfare was low because few participants reported receiving welfare (13). The subjective appraisal of whether the family was better or worse off financially than others had the lowest concordance (0.60) and only fair agreement.

**Table 2:** Frequency of concordant responses between sibling pairs on recalled measures of childhood socioeconomic position.

Measure	Pairs	Responses of each pair	N (%)	Concordance	Kappa
Father's occupation	Concordant (N=862)	Professional; Professional	94(8.2)	0.76(0.73,0.80)	0.77(0.74,0.81)
		Manager; Manager	129(11.3)		
		Technical; Technical	8(0.7)		
		Clerical; Clerical	27(2.4)		
		Sales; Sales	86(7.6)		
		Craftsman; Craftsman	178(15.6)		
		Service worker; Service worker	36(3.1)		
		Operative; Operative	157(13.8)		
		Farm worker; Farm worker	147(12.9)		
					
	Discordant (N = 276)	Professional; Manager	13(1.1)		
		Manager; Clerical	13(1.1)		
		Manager; Sales	29(2.5)		
		Manager; Craftsman	28(2.5)		
		Manager; Operative	17(1.5)		
		Sales; Operative	11(1.0)		
		Craftsman; Operative	57(5.0)		
		Operative; Farm worker	11(1.0)		
		All others	97(8.5)		
					
					
Father with professional occupation	Concordant (N=1233)	Professional; Professional	94(7.4%)	0.97(0.96,0.98)	0.82(0.77,0.89)
		Non-professional; Non-professional	1139(89.8%)		
					
	Discordant (N=35)	Professional; Non-professional	35(2.8%)		
					
					
Father with supervisory role at work	Concordant (N=895)	Supervisor; Supervisor	374(32.4)	0.77(0.75,0.80)	0.54(0.49,0.60)
		Non-supervisor; Non-supervisor	521(45.1)		
					
	Discordant (N=259)	Supervisor; Non-supervisor	259(22.5)		
					
					
Father's education level	Concordant (N=807)	0 - 8 years; 0 - 8 years	259(23.4)	0.73(0.70,0.76)	0.78(0.75,0.81)
		9-11 years; 9-11 years	49(4.4)		
		High school graduate; High school graduate	235(21.2)		
		Some college; Some college	81(7.3)		
		College graduate; College graduate	183(16.5)		
					
	Discordant (N=299)	0-8 years; 9-11 years	59(5.3)		
		0-8 years; High school graduate	49(4.4)		
		0-8 years; Some college	6(0.5)		
		0-8 years; College graduate	2(0.2)		
		9-11 years; High school graduate	63(5.7)		
		9-11 years; Some college	9(0.8)		
		High school graduate; Some college	56(5.0)		
		High school graduate; College graduate	15(1.3)		
		Some college; College graduate	40(3.6)		
					
					
Mother's education level	Concordant(N=822)	0-8 years; 0-8 years	161(13.6)	0.69(0.66,0.73)	0.73(0.70,0.76)
		9-11 years; 9-11 years	55(4.7)		
		High school graduate; High school graduate	378(32.0)		
		Some college; Some college	103(8.7)		
		College graduate; College graduate	125(10.6)		
					
	Discordant (N=359)	0-8 years; 9-11 years	74(6.3)		
		0-8 years; High school graduate	45(3.8)		
		0-8 years; Some college	1(0.08)		
		0-8 years; College graduate	0		
		9-11 years; High school graduate	91(7.7)		
		9-11 years; Some college	6(0.5)		
		9-11 years; College graduate	1(0.08)		
		High school graduate; Some college	74(6.3)		
		High school graduate; College graduate	16(1.4)		
		Some college; College graduate	51(4.3)		
					
Welfare for 6 months or longer	Concordant(N=1198)	Welfare; Welfare	29(2.3)	0.95(0.93,0.97)	0.45(0.34,0.58)
		No welfare: No welfare	1169(92.8)		
					
	Discordant(N = 62)	Welfare; No welfare	62(4.9)		
					
					
Better off financially than others	Concordant(N=700)	Better off; Better off	207(17.9)	0.60(0.57,0.64)	0.46(0.42,0.51)
		Same; Same	292(25.3)		
		Worse off; Worse off	201(17.4)		
					
	Discordant (N = 454)	Better off: Same	190(16.6)		
		Better off; Worse off	51(4.4)		
		Same; Worse off	213(18.4)		

Concordance for all measures was similar across groups stratified by age, sex, and income level (Table 3). Results were also similar between twins and siblings, suggesting that age differences between siblings did not influence concordances for those measures that might have changed over time, such as father's occupation. However, concordance for some measures varied by education level of the participants. College graduates had higher concordances for father's occupation, father's supervisory role at work, father's education level, and mother's education level than pairs with at least one member who had a high school education or less. The high concordance for father's professional occupation and for receipt of welfare did not vary with education level, nor did the relatively low concordance for subjective appraisal of whether the family was better off or worse off financially than others.

**Table 3:** Concordance of responses between sibling pairs on recalled measures of childhood socioeconomic position, by age, sex, education level, income, and twin status.

		Age < 45	Age ≥ 45	Brothers	Brother/Sister	Sisters	Less than high school	High school graduate	Some college	College graduate	Not poor	Poor	Non-twins	Twins
														
Median N (range)*		554 (500-609)	602 (552-659)	313 (288-340)	412 (397-455)	440 (393-477)	127 (102-148)	435 (405-481)	331 (304-364)	264 (254-278)	776 (714-834)	388 (354-437)	438 (411-475)	719 (650-793)
														
Father's occupation	Concordance	0.79	0.82	0.82	0.78	0.81	0.82	0.78	0.78	0.86	0.83	0.76	0.76	0.83
	Kappa	0.74	0.79	0.77	0.76	0.78	0.68	0.68	0.75	0.85	0.80	0.70	0.76	0.77
														
Father with professional occupation	Concordance	0.96	0.97	0.96	0.97	0.97	0.98	0.98	0.96	0.95	0.97	0.97	0.97	0.97
	Kappa	0.81	0.84	0.80	0.85	0.82	0.66	0.65	0.81	0.87	0.85	0.73	0.83	0.82
														
Father with supervisory role at work	Concordance	0.79	0.76	0.76	0.76	0.80	0.72	0.76	0.78	0.80	0.79	0.74	0.77	0.77
	Kappa	0.54	0.52	0.51	0.50	0.59	0.52	0.54	0.53	0.54	0.56	0.48	0.53	0.54
														
Father's education level	Concordance	0.73	0.72	0.72	0.74	0.73	0.70	0.66	0.74	0.82	0.73	0.72	0.76	0.71
	Kappa	0.77	0.76	0.77	0.79	0.78	0.66	0.66	0.78	0.83	0.78	0.77	0.82	0.76
														
Mother's education level	Concordance	0.71	0.67	0.68	0.68	0.71	0.61	0.66	0.70	0.77	0.70	0.67	0.69	0.70
	Kappa	0.74	0.70	0.71	0.72	0.75	0.56	0.65	0.70	0.80	0.73	0.70	0.72	0.73
														
Welfare for 6 months or longer	Concordance	0.95	0.94	0.94	0.95	0.95	0.91	0.93	0.96	0.99	0.95	0.93	0.96	0.94
	Kappa	0.49	0.42	0.27	0.49	0.53	0.63	0.26	0.46	0.56	0.40	0.50	0.33	0.49
														
Better off financially than others	Concordance	0.61	0.60	0.58	0.61	0.62	0.60	0.61	0.60	0.62	0.61	0.60	0.60	0.61
	Kappa	0.47	0.45	0.44	0.47	0.47	0.35	0.43	0.44	0.47	0.47	0.42	0.45	0.47

* Range of number of pairs included in analyses of different socioeconomic measures.

## Discussion

The results of this study support the validity of recalled measures of childhood socioeconomic position. These measures included not only father's occupation, supervisory role at work, and education level, which have been the measures of childhood socioeconomic position most commonly used [3], but also mother's education level and receipt of welfare payments. Concordance of recall of mother's educational level has not been reported previously. This result is important given observations that mothers' and fathers' educational levels have different and unique associations with some health outcomes [14-16]. Agreement on parent's education level was somewhat lower than that for father's occupation, but at a level similar to long-term recall of education level in other studies [17]. Father's occupation also had slightly fewer missing responses than education level, which may influence which measure is preferred. Receipt of welfare during childhood was also highly concordant, although substantial differences in childhood socioeconomic position were likely present among those whose family did not receive welfare. Concordance was lowest for the subjective appraisal of relative wealth during childhood, despite collapsing the seven response categories to three categories. This is important to note because this measure has been included in several national surveys of health and aging and has been increasingly used by researchers [18-20].

These findings support previous studies of the validity of recall of father's education level and occupation. However, concordance in our study was somewhat lower than that reported by Krieger and colleagues [8]. For example, Krieger *et al *reported percent concordance for father's education level of 0.91, compared to 0.73 in our study. Two factors may largely account for this difference. Father's education level in the study by Krieger *et al *was classified into three categories [less than high school graduate; high school graduate or some college; college graduate], while we used five categories. Re-analysis of our data using these three categories for father's education level increased the proportion of concordant responses to 0.83. More finely-graded categories provide more opportunity for discrepant responses, and therefore may have lower estimates of concordance and agreement, but may also be more useful. Participants in the study by Krieger *et al *were also more highly educated, with 28% having a high school education or less, compared to 36% in this study. We found that participants with higher levels of education level tended to have more concordant responses, particularly for parental education level, and less so for father's occupation and supervisory role at work. Low education level has been associated with poorer recall in some previous studies [21,22]. Education level was the only participant characteristic associated with differences in agreement. Agreement was similar between brothers and sisters.

The survey did not ask about residential characteristics during childhood, so recall of measures such as household crowding, plumbing, and home ownership could not be assessed. The occupation classification is specific to the United States, and these results may not be generalizable to other countries. Concordance may be higher in countries where more abbreviated occupational scales are used, such as the United Kingdom (professional; managerial and technical; skilled; partly skilled; unskilled). Data were obtained by telephone interview, which likely reduced collaboration between siblings. However, given the opportunity that a telephone interview provides for questions and clarifications, these results may not directly relate to data collected by other means, such as written questionnaires. The study included too few minorities for analyses stratified by race. In addition, the validity of recall based on the concordance of sibling responses may not replicate direct comparisons of recalled and prospectively collected data on childhood socioeconomic position.

## Conclusions

Our results indicate that adult's recall of father's occupation and parental educational levels are sufficiently valid for use in characterizing childhood socioeconomic position. However, it is important to note that agreement of recall of these measures was lower among participants with high school educations or less, which may introduce differential bias and distort associations with health outcomes. Appraisals of relative wealth during childhood also demonstrated lower concordance, as might be anticipated for a more subjective measure. Caution should be used in relying on this as the sole measure of childhood socioeconomic position, to the exclusion of other more objective measures that can be obtained equally easily.

## Abbreviations

MIDUS: National Survey of Midlife Development in the United States; U.S.: United States; N.C.: North Carolina

## Competing interests

The author declares that he has no competing interests.

## Authors' contributions

MMW conceived the study, identified the data source, designed and executed the analysis, and wrote the manuscript.

## Pre-publication history

The pre-publication history for this paper can be accessed here:

http://www.biomedcentral.com/1471-2288/11/147/prepub
